# Supplementary material for: Causal Relationship between Aging and Anorexia Nervosa: A White-Matter-Microstructure-Mediated Mendelian Randomization Analysis
Source: Biomedicines. 2024 Aug 16;12(8):1874. doi: 10.3390/biomedicines12081874 (PMC11351342; doi:10.3390/biomedicines12081874)
Supplement: Supplementary file 1 [file biomedicines-12-01874-s001.zip › Supplementary Figure/Figure S4/Singe SNP plot of MR analysis for TL to ubm-b-1926.pdf]

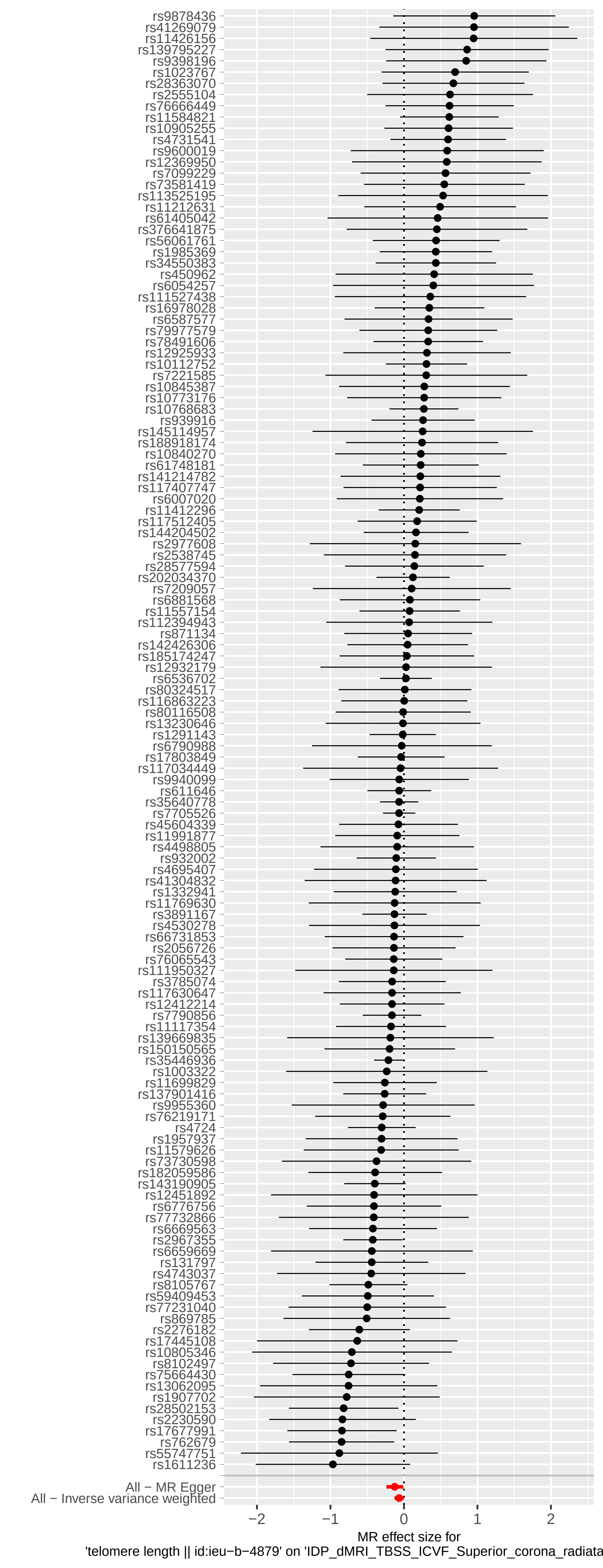

rs9878436
rs41269079
rs11426156
rs139795227
rs9398196
rs1023767
rs28363070
rs2555104
rs76666449
rs11584821
rs10905255
rs4731541
rs9600019
rs12369950
rs7099229
rs73581419
rs113525195
rs11212631
rs61405042
rs376641875
rs56061761
rs1985369
rs34550383
rs450962
rs6054257
rs111527438
rs16978028
rs6587577
rs79977579
rs78491606
rs12925933
rs10112752
rs7221585
rs10845387
rs10773176
rs10768683
rs939916
rs145114957
rs188918174
rs10840270
rs61748181
rs141214782
rs117407747
rs6007020
rs11412296
rs117512405
rs144204502
rs2977608
rs2538745
rs28577594
rs202034370
rs7209057
rs6881568
rs11557154
rs112394943
rs871134
rs142426306
rs185174247
rs12932179
rs6536702
rs80324517
rs116863223
rs80116508
rs13230646
rs1291143
rs6790988
rs17803849
rs117034449
rs9940099
rs611646
rs35640778
rs7705526
rs45604339
rs11991877
rs4498805
rs932002
rs4695407
rs41304832
rs1332941
rs11769630
rs3891167
rs4530278
rs66731853
rs2056726
rs76065543
rs111950327
rs3785074
rs117630647
rs12412214
rs7790856
rs11117354
rs139669835
rs150150565
rs35446936
rs1003322
rs11699829
rs137901416
rs9955360
rs76219171
rs4724
rs1957937
rs11579626
rs73730598
rs182059586
rs143190905
rs12451892
rs6776756
rs77732866
rs6669563
rs2967355
rs6659669
rs131797
rs4743037
rs8105767
rs59409453
rs77231040
rs869785
rs2276182
rs17445108
rs10805346
rs8102497
rs75664430
rs13062095
rs1907702
rs28502153
rs2230590
rs17677991
rs762679
rs55747751
rs1611236
All − MR Egger
All − Inverse variance weighted
−2
−1
0
1
2
MR effect size for
'telomere length || id:ieu−b−4879' on 'IDP_dMRI_TBSS_ICVF_Superior_corona_radiata
